# Pathogenic variant detection rate by whole exome sequencing in Thai patients with biopsy-proven focal segmental glomerulosclerosis

**DOI:** 10.1038/s41598-022-26291-y

**Published:** 2023-01-16

**Authors:** Suramath Isaranuwatchai, Ankanee Chanakul, Chupong Ittiwut, Rungnapa Ittiwut, Chalurmpon Srichomthong, Vorasuk Shotelersuk, Kanya Suphapeetiporn, Kearkiat Praditpornsilpa

**Affiliations:** 1 Division of Nephrology, Department of Internal Medicine, Faculty of Medicine, Chulalongkorn University, Bangkok, Thailand; 2 Division of Nephrology, Department of Internal Medicine, Chulabhorn Hospital, Chulabhorn Royal Academy, Bangkok, Thailand; 3 Division of Nephrology, Department of Pediatrics, Faculty of Medicine, Chulalongkorn University, Bangkok, Thailand; 4 Division of Medical Genetics and Metabolism, Department of Pediatrics, Center of Excellence for Medical Genomics, Medical Genomics Cluster, Faculty of Medicine, Chulalongkorn University, Bangkok, 10330 Thailand; 5 Excellence Center for Genomics and Precision Medicine, King Chulalongkorn Memorial Hospital, the Thai Red Cross Society, Bangkok, Thailand

**Keywords:** Genomics, Kidney diseases

## Abstract

The spectra of underlying genetic variants for various clinical entities including focal segmental glomerulosclerosis (FSGS) vary among different populations. Here we described the clinical and genetic characteristics of biopsy-proven FSGS patients in Thailand. Patients with FSGS pathology, without secondary causes, were included in our study. Clinical laboratory and pathological data were collected. Whole-exome sequencing (WES) was subsequently performed. 53 unrelated FSGS patients were recruited. 35 patients were adults (66.0%), and 51 patients were sporadic cases (96.2%). Clinical diagnosis before kidney biopsy was steroid-resistant nephrotic syndrome (SRNS) in 58.5%, and proteinuric chronic kidney disease in 32.1%. Using WES, disease-associated pathogenic/likely pathogenic (P/LP) variants could be identified in six patients including the two familial cases, making the P/LP detection rate of 11.3% (6/53). Of these six patients, two patients harbored novel variants with one in the *COL4A4* gene and one in the *MAFB* gene. Four other patients carried previously reported variants in the *CLCN5*, *LMX1B,* and *COL4A4* genes. Four of these patients (4/6) received immunosuppressive medications as a treatment for primary FSGS before genetic diagnosis. All four did not respond to the medications, emphasizing the importance of genetic testing to avoid unnecessary treatment. Notably, the mutation detection rates in adult and pediatric patients were almost identical, at 11.4% and 11.1%, respectively. In conclusion, the overall P/LP variant detection rate by WES in biopsy-proven FSGS patients was 11.3%. The most identified variants were in *COL4A4*. In addition, three novel variants associated with FSGS were detected.

## Introduction

Focal segmental glomerulosclerosis (FSGS) is one of the most common glomerular diseases in Thailand^1^ and worldwide^2^. Traditionally, FSGS was classified into primary FSGS and secondary FSGS^3^. Nephrologists usually differentiate primary and secondary FSGS by finding the secondary cause of FSGS namely obesity, obstructive nephropathy, human immunodeficiency virus (HIV) infection, and drug-induced FSGS. Recently, pathogenic variants in genes encoding proteins involving podocyte slit diaphragms, podocyte cytoskeletons, and glomerular basement membrane (GBM) were found in 11–43% of FSGS patients^4–6^. These FSGS patients were classified as genetic FSGS. The genetic contribution of FSGS has been underrecognized and underdiagnosis due to high cost, unavailability of genetic testing and unawareness of clinicians. However, knowing the underlying genetic defects could prevent many of these patients from unnecessary immunosuppressive medications.

The selection of patients with FSGS for genetic testing can be challenging. According to Current Kidney Disease: Improving Global Outcomes (KDIGO) guideline, genetic testing is not recommended in all FSGS patients^7^. The guideline suggests that genetic testing could be considered in FSGS patients with a strong family history or with syndromal features. Genetic testing might also be useful to determine the risk of FSGS recurrence after kidney transplantation. Other studies suggested genetic testing might be appropriate in other settings such as patients with steroid-resistant nephrotic syndrome (SRNS)^8,9^. Further studies of FSGS are in need for evaluating the clinical utility of genetic testing in appropriate subgroups of FSGS patients.

Here, we conducted a study to characterize the clinical and genetic features of biopsy-proven FSGS patients, using whole-exome sequencing (WES). This study is the first to evaluate the genetic etiology of patients with FSGS in the Thai population.

## Methods

### Patients and recruitment

We reviewed the renal pathology report at King Chulalongkorn Memorial Hospital from January 2000 to December 2020. The inclusion criteria were patients who had FSGS on kidney pathology. The diagnosis of FSGS was made by the presence of segmental sclerosis and or glomerular adhesion to Bowman’s capsule presented in light microscopy (LM) or immunofluorescent (IF). Exclusion criteria were patients with secondary causes of FSGS, including obstructive uropathy, lupus nephritis, reduced kidney mass, congenital anomaly of kidney and urinary tract, HIV infection, and parvovirus B19 infection. Patients receiving medications causing FSGS including gold, penicillamine, and pamidronate were also excluded. The renal pathology of FSGS in LM was classified by Columbia classification^10^. Electron microscopy (EM) was also reviewed but not mandatory for recruitment for this study. Patients were contacted by telephone and at the renal clinic for informed consent. All participants then had given written informed consent for this study. The Ethics Committee of Faculty of Medicine, Chulalongkorn University, approved the protocol of this study (Med Chula IRB no.1516/2562) in compliance with the International guidelines for human research protection as Declaration of Helsinki and International Conference on Harmonization in Good Clinical Practice.

### Data collection

Clinical characteristics and renal pathology were reviewed. The patients’ clinical manifestations were classified into four main categories: asymptomatic proteinuria, steroid-sensitive nephrotic syndrome (SSNS), steroid-resistant nephrotic syndrome (SRNS) defined as nephrotic syndrome patients who did not respond to steroid treatment for 4 weeks in children and 16 weeks in adults^7^, and proteinuric chronic kidney disease (CKD).

### Genetic study

Genomic DNA was extracted from peripheral blood leucocytes. The DNA sample was prepared as an Illumina sequencing library. The sequencing libraries were enriched by TruSeq® Exome Kit (Illumina Inc., Illumina, San Diago, CA) and were sequenced onto NextSeq 500 System (Illumina, San Diago, CA). Sequence reads were mapped against UCSC hg19 using Burrows-Wheeler Alignment (BWA) software. Variant calling was performed using GATK with *HaplotypeCaller.*

### Variant interpretation

**Figure 1:**
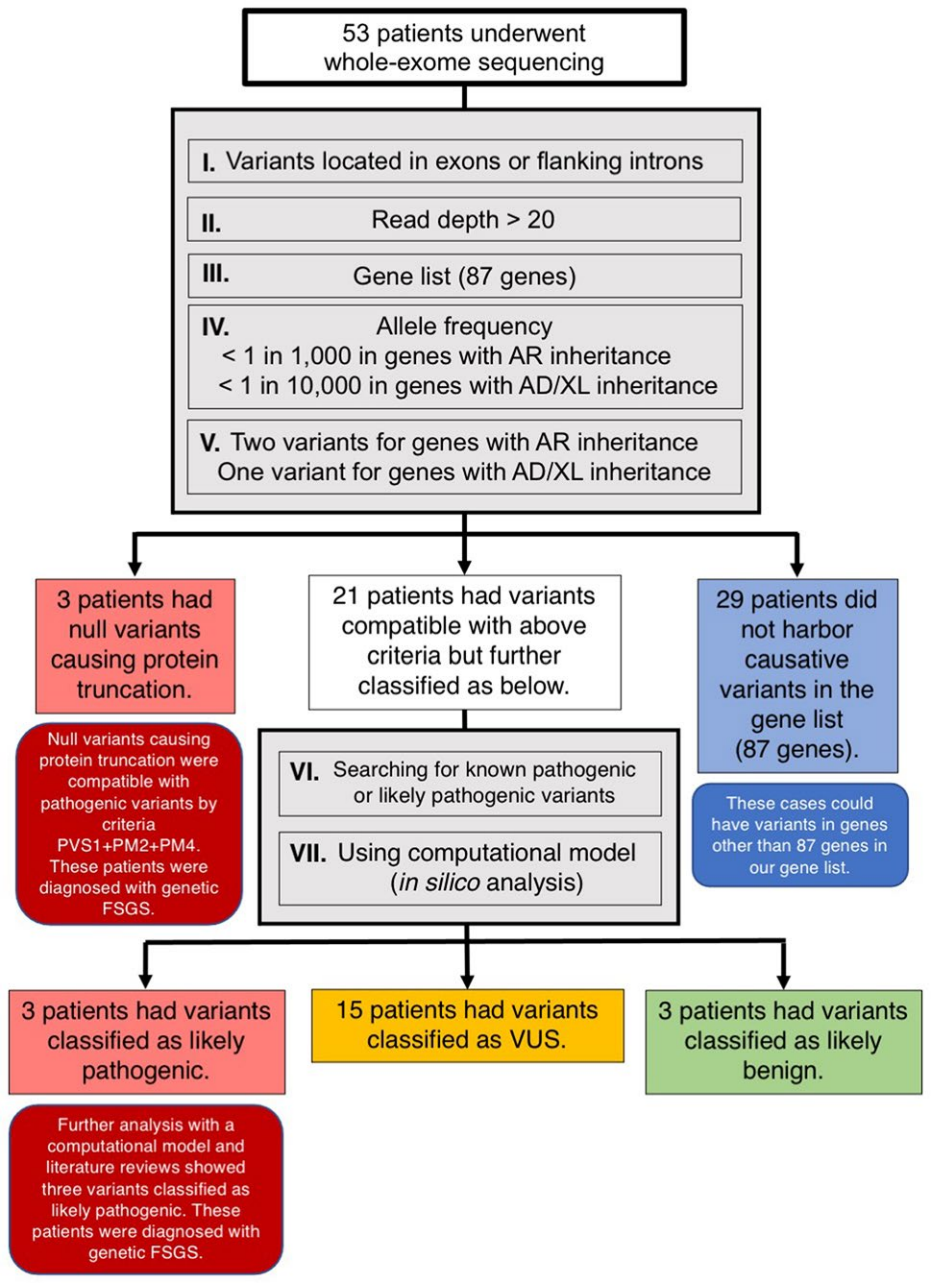
Variant interpretation. The algorithm for variant interpretation: Five criteria (I–V) were applied including (I.) coding consequences, (II.) with read depth more than 20, (III.) within the gene list with 87 genes associated with FSGS, (IV.) with allele frequency of less than 1 in 1000 in genes with AR inheritance and less than 1:10,000 in genes with AD or XL inheritance, and (V.) one variant in genes with AD/XL inheritance but two variants in genes with AR inheritance. *AD* autosomal dominant, *AR* autosomal recessive, *FSGS* focal segmental glomerulosclerosis, *VUS* variant of uncertain significance, *XL* X-linked.

Golden Helix Genome Browser (Nagoya, Aichi-pref., Japan) and BaseSpace Variant Interpreter (Illumina, San Diago, CA) were used for data analysis. We used the gene list to screen variants identified by WES. Eighty-seven genes were previously identified as causative genes or genes associated with FSGS, shown in supplementary table S1. Phenocopy disease genes, responsible for diseases resembling FSGS shown by renal histopathology such as Alport’s syndrome or Fabry’s disease, were included in this gene list. All candidate variants were evaluated by clinical geneticists and nephrologists and classified according to American College of Medical Genetics and Genomics (ACMG) interpretation guidelines^11^. For the *COL4A3*, *COL4A4,* and *COL4A5* genes, we used recommendations for variant interpretation as previously suggested by expert consensus^12,13^. Our detailed algorithm for variant interpretation is shown in Fig. 1.

## Results

### Clinicopathological characteristics

53 patients were included in our study. Clinical, laboratory, and pathological characteristics of FSGS patients in our cohort are shown in Table 1 and supplementary table S2. Two cases had a family history of kidney diseases (2/53 = 3.8%). Only one patient had extra-renal manifestation compatible with Noonan syndrome. 52.8% percent (28/53) of the patients were male. Two-thirds (35/53 = 66.0%) were adult patients older than 18 years old. The most common clinical diagnosis/syndrome was SRNS (31/53 = 58.5%), followed by proteinuric CKD (17/53 = 32.1%), and SSNS (5/53 = 9.4%). Most of the patients (40/53 = 75.5%) received immunosuppressive medications. With an average follow-up time of 9.5 years, most of them still had proteinuria (36/53 = 67.9%). Of 31 patients in the SRNS group, only 6 patients (6/31 = 19.4%) responded to immunosuppressive therapy. Among 12 patients (22.6%) who reached end-stage renal disease (ESRD), only two (2/12 = 16.7%) underwent kidney transplantation. No recurrence appeared after kidney transplantation in both patients.

**Table 1:** Clinical and pathological characteristics of Thai patients with FSGS.

Categories	Number of patients (%)
**Clinical characteristics**	
**Sex**	
Male	28/53 (52.8%)
**Age at kidney biopsy**	
Age 1–5 years	6/53 (11.3%)
Age 6–18 years	12/53 (22.6%)
Age 19–45 years	19/53 (35.9%)
Age 45–60 years	13/53 (24.5%)
Age > 60 years	3/53 (5.7%)
**Family history of kidney diseases**	
Positive	2/53 (3.8%)
Extra-renal syndromic manifestation	
Positive	1/53 (1.9%)
**Clinical diagnosis/syndrome**	
SSNS	5/53 (9.4%)
SRNS	31/53 (58.5%)
Proteinuric CKD	17/53 (32.1%)
Immunosuppressive medications received	40/53 (75.5%)
Mean time of follow-up	9.5 years
**Current status**	
No proteinuria and normal creatinine	4/53 (7.5%)
Proteinuric CKD	36/53 (67.9%)
ESRD	12/53 (22.7%)
Dead	1/53 (1.9%)
Median time from FSGS diagnosis to ESRD	8 years
*Pathological characteristics (Light microscopy)*	
**Columbia classification of FSGS**	
Tip lesion	11/53 (20.7%)
Hilar/Perihilar lesion	4/53 (7.5%)
Cellular variant	2/53 (3.8%)
Collapsing variant	2/53 (3.8%)
Not otherwise specified (NOS)	34/53 (64.2%)
*Pathological characteristics (Immunofluorescent study)*	
Negative or non-specific IF staining	17/42 (40.5%)
Only segmental IgM and/or C3 staining	11/42 (26.2%)
Mesangial IgM and/or C3 staining	13/42 (30.9%)
Others*	1/42 (2.4%)
*Pathological characteristics (Electron microscopy)*	
**Podocyte foot process effacement**	
No	0/18 (0%)
Focal	12/18 (66.7%)
Diffused	6/18 (33.3%)
**Microvillous transformation**	
No	2/18 (11.1%)
Focal	11/18 (61.1%)
Diffused	5/18 (27.8%)
Irregular GBM	10/18 (55.6%)

*CKD* chronic kidney disease, *ESRD* end-stage renal disease, *FSGS* focal segmental glomerulosclerosis, *GBM* glomerular basement membrane, *IF* immunofluorescent study, *IgM* immunoglobulin M, *SRNS* steroid-resistant nephrotic syndrome, *SSNS* steroid-sensitive nephrotic syndrome.

*One other case had trace coarse granular staining of IgM, kappa and lambda along capillary loop with focal linear C3 staining along Bowman’s capsule.

The most common FSGS subtype in this cohort according to Columbia classification was not-otherwise specified (NOS) (34/53 = 64.2%), followed by tip lesion (11/53 = 20.7%), hilar or perihilar lesion (4/53 = 7.5%), cellular variant (2/53 = 3.8%) and collapsing variant (2/53 = 3.8%). IF was obtained in 42 patients. The majority had negative IF staining (17/42 = 40.5%). Mesangial IF staining and segmental IF staining of IgM and/or C3 were present in 13 cases (13/42 = 30.9%) and 11 cases (11/42 = 26.2%), respectively. EM was obtained in 18 patients. All patients with EM had podocyte foot process effacement (FPE). There were 12 patients (12/18 = 66.7%) with focal FPE and six patients (6/18 = 33.3%) with diffused FPE. Microvillous transformation was identified in 13 patients (13/18 = 72.2%). Irregular GBM was found in ten patients (10/18 = 55.6%).

### Genetic results

**Figure 2:**
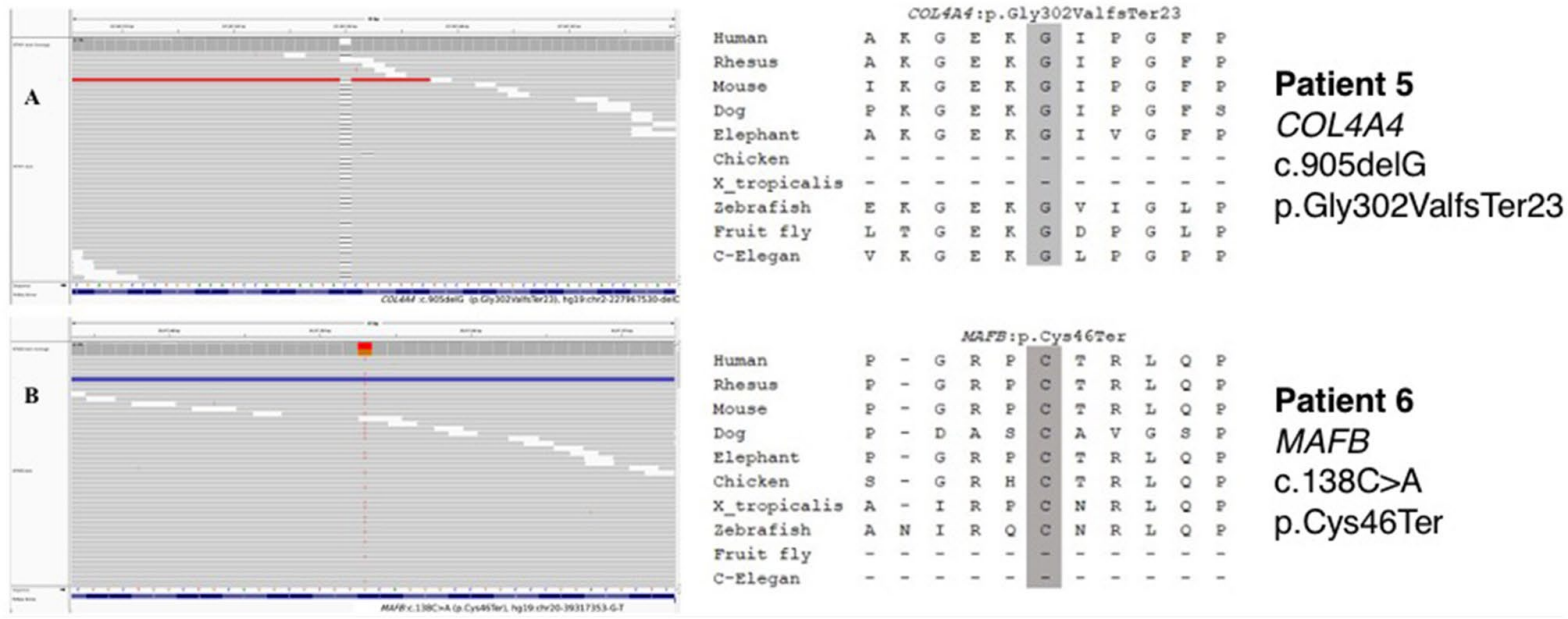
Screenshots of WES alignments of three novel variants and sequence alignment of partial amino acid sequences of *COL4A4* (**A**) and *MAFB* (**B**) from various species.

Of the 53 unrelated cases, 52 and one families underwent WES using only one member (the proband; singleton), three members (the proband and parents; trio), respectively. Overall, six of 53 patients had pathogenic/likely pathogenic (P/LP) variants (6/53 = 11.3%) as defined by ACMG criteria. Of the six variants identified, three were novel (Table 2). These variants included three missense, two nonsense, and one frameshift. Detailed information of the causal variants is shown in Table 2. Fifteen patients harbored variants of uncertain significance (VUS) (Supplementary Table S4). The algorithm and results of the genetic analysis are shown in Fig. 1. Sequence alignment and conservation of the novel variants are shown in Fig. 2.

**Table 2:** Clinical, laboratory, pathologic characteristics, and genetic variants of patients with pathogenic or likely pathogenic mutations.

	Patient 1	Patient 2	Patient 3	Patient 4	Patient 5	Patient 6
**Clinical characteristics**						
Age at kidney biopsy (years)	4	9	52	52	38	47
Sex	Male	Male	Female	Female	Female	Male
Clinical diagnosis/syndrome	Asymptomatic proteinuria	SRNS	Proteinuric CKD	SRNS	SRNS	Proteinuric CKD
Family history of kidney diseases	No	Yes	No	No	Yes	No
Extra-renal syndromic manifestation	None	None	None	None	None	None
Immunosuppressive medication received	Prednisolone	Prednisolone, CNIs	None	Prednisolone	Prednisolone	None
Current status	Proteinuric CKD	ESRD on PD	Proteinuric CKD	Proteinuric CKD	ESRD on HD	ESRD on PD
Age at ESRD (years)	N/A	15	N/A	N/A	48	61
**Laboratory characteristics**						
Serum creatinine at biopsy (mg/dL)	0.19	0.40	1.20	1.70	2.09	3.20
Proteinuria at biopsy (gm/day)	4.50 (on ACEI)	2.40 (on ACEI)	2.15 (on ACEI)	2.60 (on ARB)	4.07 (on ARB)	5.04 (No ACEI/ARB)
Current serum creatinine (mg/dL)	0.30	N/A	1.58	3.87	N/A	N/A
Current proteinuria (gm/day)	5.42 (on ACEI)	N/A	1.38 (on ARB)	1.71 (on ARB)	N/A	N/A
**Pathological characteristics**						
Columbia classification of FSGS (LM)	NOS	NOS	Hilar	Hilar	NOS	NOS
Immunofluorescent staining	No data	Negative	Segmental IgM staining	Negative	Segmental IgM and C3 staining	No data
Podocyte foot process effacement (EM)	Focal	No data	No data	Focal	No data	Focal
Microvillous transformation (EM)	Yes	No data	No data	Yes	No data	No
Irregular GBM (EM)	No	No data	No data	No	No data	No
**Genetic variants**						
Gene	*CLCN5*	*LMX1B*	*COL4A4*	*COL4A4*	*COL4A4*	*MAFB*
Inheritance	AD	AD	AD	AD	AD	AD
Chromosome (HG19)	chrX:49855147	chr9:129455598	chr2:227942792	chr2:227919418	chr2:227967530	chr20:39317353
NM	NM_001127898.3	NM_001174146.1	NM_000092.4	NM_000092.4	NM_000092.4	NM_005461.4
Type of mutation	Nonsense	Missense	Missense	Missense	Frameshift indels	Nonsense
Known/novel	Known^14^	Known^15^	Known^16,17^	Known^18,19^	Novel	Novel
Variant	c.2119C > T (p.Arg707Ter)	c.737G > A (p.Arg246Gln)	c.1805G > A (p.Gly602Glu)	c.2752G > A (p.Gly918Arg)	c.905delG (p.Gly302ValfsTer23)	c.138C > A (p.Cys46Ter)
Variance conclusion	Pathogenic	Likely Pathogenic	Likely pathogenic	Likely pathogenic	Likely pathogenic	Likely pathogenic
ACMG criteria	PVS1 + PM2 + PM4	PS3 + PM2 + PP3	PM1 + PM2 + PP3 + PP5	PM1 + PM2 + PP3 + PP5	PVS1 + PM2	PVS1 + PM2

*ACEI* angiotensin-converting enzyme inhibitors, *ACMG* American College of Medical Genetics and Genomics, *AD* autosomal dominant, *ARB* angiotensin receptor blockers, *CKD* chronic kidney disease, *EM* electron microscopy, *ESRD* end-stage renal disease, *IgM* immunoglobulin M, *FSGS* focal segmental glomerulosclerosis, *GBM* glomerular basement membrane, *HD* hemodialysis, *NOS* not otherwise specified, *PD* peritoneal dialysis, *SRNS* steroid-resistant nephrotic syndrome.

In subgroup analysis, the mutation detection rate in adult patients was 11.4% (4/35). The mutation detection rate in pediatric patients was 11.1% (2/18). All patients with a family history of renal diseases (2/2 = 100%) had P/LP variants, one with a novel variant, c.905delG (p.Gly302ValfsTer23) in *COL4A4*, and the other with a known variant, c.737G > A (p.Arg246Gln) in *LMX1B*. In 51 sporadic cases, the mutation detection rate was 7.8% (2/51). Among patients presenting with SRNS, the mutation detection rate was 6.5% (2/31). Among patients presenting with SSNS, no disease-associated variants were identified. Of the six patients with P/LP variants, three (50%) had disease-associated variants in the *COL4A4* gene. There were some characteristic features in renal pathology associated with genetic FSGS. All patients with genetic FSGS had hilar or NOS lesions in LM. None of them had tip lesion or cellular or collapsing variants. All three genetic FSGS patients with EM results had focal FPE with microvillous transformation (Table 2).

The median age of disease onset and of ESRD of these 53 cases was 33 and 47 years, respectively. Among FSGS patients with disease-associated variants, the median age of disease onset and of ESRD was 42.5 and 48 years, respectively.

Clinical and pathological characteristics of patients with P/LP variants are shown in Table 2 and supplementary table S3. Patient 1 presented with a febrile urinary tract infection at the age of one year and a history of passing stones at four years of age. Investigations revealed nephrotic range proteinuria, hypercalciuria, and nephrocalcinosis. The urinary concentration of low-molecular-weight proteins and urinary beta-2-microglobulin were not measured. Given the male gender, laboratory data, and the absence of signs of other diseases, Dent disease was primarily considered. He was briefly treated with high-dose prednisolone with an absence of response. He underwent a renal biopsy and was diagnosed with FSGS, NOS with focal FPE in EM. To establish a definitive molecular diagnosis, exome sequencing was performed and a known hemizygous nonsense variant (c.2119C > T, p.Arg707Ter) in the CLCN5 gene was identified, resulting in a diagnosis of Dent disease type 1^14^. Currently, he had good renal function and his proteinuria was controlled by an angiotensin-converting enzyme inhibitor (ACEI).

Patient 2 presented with SRNS and did not respond to prednisolone, cyclosporin and tacrolimus. His renal biopsy showed FSGS, NOS with negative IF staining. His renal function rapidly declined until he reached ESRD just six years after presentation. His father also had kidney transplantation (KT) at the age of 37. The pedigree was shown in supplementary Fig. S1A. Both were found to have a known missense variant (c.737G > A, p.Arg246Gln) in the *LMX1B* gene^15^. Currently, patient 2 underwent peritoneal dialysis awaiting KT.

Patient 3 presented with proteinuric CKD. Her urinalysis showed microscopic hematuria without history of gross hematuria. She did not receive any immunosuppressive medication but was treated with an angiotensin receptor blocker (ARB). Her kidney biopsy showed hilar FSGS with segmental IgM staining in IF. WES revealed a known missense variant (c.1805G > A, p.Gly602Glu) in *COL4A4*^16,17^. After 17 years of follow-up, she was still at CKD stage 3b.

Patient 4 had SRNS, which did not respond to prednisolone. She did not have a history of episodic macroscopic hematuria, but her urinalysis showed microscopic hematuria. Her kidney biopsy showed hilar FSGS with negative IF staining and focal FPE in EM. WES revealed a known missense variant in *COL4A4* (c.2752G > A, p.Gly918Arg)^18,19^. After eight years of follow-up, she was at CKD stage 4 and treated with ARB.

Patient 5 presented with SRNS and did not respond to prednisolone. Her urinalysis showed microscopic hematuria without history of gross hematuria. Her renal function gradually declined until she reached ESRD in ten years. She also had a family history of kidney disease, with her pedigree shown in supplementary Fig. S1B. Her younger brother had a history of dialysis, but he died before patient 5 was offered genetic testing. WES revealed a novel deletion in *COL4A4*, leading to frameshift and premature stop codon (c.905delG, p.Gly302ValfsTer32). Now she underwent hemodialysis, waiting for KT.

Patient 6 presented to our clinic with CKD and proteinuria of 5.04 g/day. He did not receive any immunosuppressive medication. His renal biopsy showed FSGS, NOS with focal FPE in EM. He was found to have a novel nonsense variant (c.138C > A, p.Cys46Ter) in the *MAFB* gene. His renal function gradually declined until he reached ESRD in 13 years. Currently, he underwent peritoneal dialysis, also waiting for KT.

## Discussion

In this study, we analyzed the clinical and genetic data of 53 biopsy-proven FSGS patients. Using whole exome sequencing, as a first-tier diagnostic test, P/LP variants were identified in six patients (11.3%). All patients with a family history of renal diseases (2/2 = 100%) had P/LP variants. In 51 sporadic cases, the mutation detection rate was 7.8% (4/51).

40 patients in our cohort (75.5%) received immunosuppressive medications as a treatment of presumed primary FSGS. Only five patients (9.4%) responded to high-dose corticosteroid, which were categorized as SSNS. None of them were found to harbor causative variants. These findings also supported previous recommendations not to do genetic testing in SSNS patients^9^. 31 of them (58.5%) met the criteria of SRNS. However, causative variants were identified in only two (6.5%). Two of these patients with genetic FSGS (2/6 = 33.3%) did not receive any immunosuppressive medication. This could occur because nephrologists decided not to prescribe immunosuppressive medications in 24.5% of our cohort even though the patients had FSGS with no apparent secondary causes. Our practice was in line with the new 2021 KDIGO guideline for glomerular diseases^7^. We applied clinicopathological parameters to select only some patients to be treated with corticosteroids, including the clinical presentation of nephrotic syndrome and diffused podocyte foot process effacement in EM.

Three of our patients (3/6 = 50.0%) had a disease-associated variant in the *COL4A4* gene. This finding was in line with previous studies^4,20^ demonstrating that the *COL4A3/4/5* were among the most common genes responsible for FSGS. The *COL4* genes (*COL4A3*, *COL4A4*, and *COL4A5*), which encode collagen type 4, are essential for normal GBM. Mutations in these genes have been implicated as the cause of Alport syndrome. In addition, several studies have demonstrated that *COL4A3-5* mutations are associated with FSGS pathology. We identified three disease-associated variants in the *COL4A4* gene in unrelated patients. All were found at the conserved glycine residue (Table 2). Glycine residues in inter-collagenous domains were highly conserved from *H sapiens* (humans) to *X tropicalis* (frogs) and critical for collagens to be folded and function as normal GBM^12^. The missense mutations at the glycine residue are increasingly reported to be likely pathogenic without a functional study. However, some of these missense mutations were re-classifies as benign after functional studies were performed to evaluate the molecular effect^21^. Therefore, further studies to confirm disease-variant association and elucidate the underlying mechanism are required.

Our findings combined with reviewed data from previous studies (Table 3) emphasized the role of genetic testing on the management of FSGS patients. The main indications for genetic testing include extrarenal syndromic manifestations, history of consanguinity, and family history of renal diseases. The positive rate among these patients is very high. It should be noted to clinicians that a family history of dialysis or CKD could be a clue for genetic testing as discussed in patients 2 and 5 in our study. It should be noted that the clinical manifestations can be diverse and intrafamilial, and interfamilial variabilities have been described in families with genetic FSGS^22^.

**Table 3:** Comparison between our FSGS cohort and previous studies.

	Our cohort	Santin CJASN 2011	Sadowski JASN 2015	Gast NDT 2016	Bierzynska KI 2017	Sen JMG 2017	Warejko CJASN 2018	Gribouval KI 2018	Yao CJASN 2019	Ammar JHG 2021	Braunisch EJHG 2021	Miao Mayo 2021
References		^8^	^25^	^4^	^26^	^27^	^28^	^29^	^5^	^30^	^31^	^6^
**Cohort characteristics**												
Total patients	53	125	2016	81	187	302	335	135	193	21	50	49
Number of family	53	110	1783	76	N/A	N/A	300	135	179	7	24	N/A
Number of family with history of renal diseases	2/53 (3.8%)	24/110 (21.8%)	N/A	24/76 (31.6%)	22/187 (11.8%)	58/183 (31.7%)	126/300 (42.0%)	0/135 (0%)	29/179 (16.2%)	7/7 (100%)	N/A	20/48 (41.7%)
Asian	53/53 (100%)	N/A	159/1783 (8.9%)	1/76 (1.3%)	24/187 (12.8%)	28/150 (18.7%)	136/300 (45.3%)	N/A	24/193 (12.4%)	0/21 (0%)	N/A	N/A
Consanguinity	0/53 (0%)	7/110 (6.4%)	372/1783 (20.9%)	N/A	13/187 (7.0%)	17/141 (12.1%)	146/300 (48.6%)	0/135 (0%)	N/A	6/7 (85.7%)	2/24 (8.3%)	N/A
Adult patients (age > 18 years)	35/53 (66.1%)	48/110 (43.6%)	28/1783 (1.6%)	70/81 (86.4%)	0/187 (0%)	62/302 (20.5%)	8/335 (2.4%)	135/135 (100%)	N/A	N/A	24/24 (100%) In index cases	49/49 (100%)
FSGS pathology	53/53 (100%)	108/125 (86.4%)	N/A	73/81 (90.1%)	98/187 (52.4%)	115/160 (71.9%)	153/223 (58.3%)	100/135 (74.1%)	148/148 (100%)	11/21 (52.4%)	N/A	49/49 (100%)
SRNS	30/53 (56.6%)	78/125 (62.4%)	1783/1783 (100%)	N/A	181/187 (96.8%)	255/302 (84.4%)	205/300 (68.3%)	N/A	N/A	21/21 (100%)	N/A	N/A
**Mutation detection rate**												
Overall	6/53 (11.3%)	37/110 (33.6%)	526/1783 (29.5%)	10/76 (13.2%)	49/187 (26.2%)	71/302 (23.5%)	74/300 (24.7%)	16/135 (11.8%)	20/179 (11.2%)	7/7 (100%)	7/24 (29.2%)	21/49 (42.9%)
Among patients with FSGS pathology	6/53 (11.3%)	20/108 (18.5%)	N/A	9/75 (12.0%) FSGS/SRNS combined	N/A	N/A	40/153 (26.1%)	14/100 (14.0%)	20/179 (11.2%)	N/A	N/A	21/49 (42.9%)
Among patients with SRNS	2/31 (6.5%)	18/78 (23.1%)	526/1783 (29.5%)		48/181 (26.5%)	54/255 (21.2%)	57/205 (27.8%)	N/A	N/A	21/21 (100%)	N/A	6/13 (42.6%)
Among adult patients (age > 18 years)	4/35 (11.4%)	7/48 (14.6%)	6/28 (21.4%)	9/69 (13.0%)	N/A	19/62 (30.6%)	2/8 (25.0%)	16/135 (11.8%)	N/A	N/A	N/A	21/49 (42.9%)

*ESRD* end-stage renal disease, *FSGS* focal segmental glomerulosclerosis, *N/A* not available, *SRNS* steroid-resistant nephrotic syndrome.

After FSGS patients progress to CKD, genetic testing may be of benefit when the patients undergo KT. Recurrence of FSGS in the transplanted kidney is a troublesome condition that requires intense treatment with plasmapheresis and immunosuppression. Genetic FSGS is known to have a very low recurrence rate, reaching 0% in some studies; however, recurrence can be as high as 50% in primary FSGS^23,24^. Knowing genetic causes before KT will be beneficial in management after KT since recurrent FSGS can be presented as early as the first day after KT. In many countries including Thailand, to prevent organ trafficking, living-donor KT is restricted only to family members. Genetic diagnosis will be a very important diagnostic tool before KT in the dis-incentivizing countries with living-donors limited to family members. Although genetic testing might result in limited potential living-donors, taking one kidney from a donor with the variant causing FSGS could make a donor turn into CKD oneself. Therefore, genetic testing could provide important data for both donors and recipients before making a decision to undergo KT.

There are some limitations in our study. The small pediatric sample size might explain the lower mutation detection rate in our study. Several variants were classified as VUS when data were from singleton WES. A large-scale study with genetic testing in family members could add more data in this area.

In conclusion, a monogenic cause was identified in 11.3% of FSGS patients. Most of the causative variants were found in the *COL4A4* genes. This is the first study to evaluate the genetic etiology of patients with FSGS in the Thai population. Our study also highlights the importance of WES in FSGS patients who do not respond to immunosuppressive treatment, have extra-renal syndromic manifestations, and have a family history of renal disease or consanguinity or will undergo kidney transplantation.

## Supplementary Information

Supplementary Information.

## Data Availability

The datasets used and/or analysed during the current study available from the corresponding author on reasonable request.
