# Supplementary material for: Pathogenic variant detection rate by whole exome sequencing in Thai patients with biopsy-proven focal segmental glomerulosclerosis
Source: Sci Rep. 2023 Jan 16;13:805. doi: 10.1038/s41598-022-26291-y (PMC9842604; doi:10.1038/s41598-022-26291-y)
Supplement: Supplementary file 1 — Supplementary Information. [file 41598_2022_26291_MOESM1_ESM.docx]

**Supplementary materials**

|  | **Page** |
| --- | --- |
| **Supplementary Table S1:** Gene list | **2** |
| **Supplementary Table S2:** Detailed Clinical, laboratory, and pathological characteristics of FSGS patients in our cohort | **7** |
| **Supplementary Table S3:** Variant interpretation of pathogenic or likely pathogenic variants | **8** |
| **Supplementary Table S4:** Clinical characteristics and variant interpretation of patients with variants of uncertain significance (VUS) | **9** |
| **Supplementary Figure S1:** Pedigrees of two patients with a family history of renal diseases | **10** |

**Supplementary Table S1: Gene list**

| **No** | **Gene** | **Location** | **Inh.** | **Protein** | **Clinical syndrome** | **Reference** |
| --- | --- | --- | --- | --- | --- | --- |
| 1 | *ACSL4* | Xq23 | XL | acyl-CoA synthetase long chain family member 4 | X-linked intellectual development disorder | 1 |
| 2 | *ACTN4* | 19q13.2 | AD | Alpha actinin 4 | FSGS/SRNS | 1 |
| 3 | *ADCK4* | 19q13.2 | AR | Aarf domain containing kinase 4 (Coenzyme Q8B) | NS, childhood, adult, seizures | 4 |
| 4 | *AGXT* | 2q37.3 | AR | Alanine-glyoxylate aminotransferase | Primary hyperoxaluria type 1 | 9 |
| 5 | *ALG1* | 16p13.3 | AR | Asparagine-linked glycosylation 1 | Congenital disorder of glycosylation, CNS | 1 |
| 6 | *ALG13* | Xq23 | XL | UDP-N-acetylglucosaminyltranstease subunit | Congenital disorder of glycosylation, CNS | 6 |
| 7 | *ALMS1* | 2p13.1 | AR | Centrosome and basal body associated protein ALMS1 | Alstrom syndrome | 2 |
| 8 | *ANLN* | 7p14.2 | AD | Anillin actin-binding protein | FSGS, adult | 5 |
| 9 | *APOE* | 19q13.32 | AR | apolipoprotein E | Hyperlipoproteinemia type 3 | 1 |
| 10 | *APOL1* | 22q12.3 | AR | Apolipoprotein L-1 | FSGS | 1 |
| 11 | *ARHGAP24* | 4q21.23 | AD | Rho-GTPase activating protein | FSGS, adult | 1 |
| 12 | *ARHGDIA* | 17q25.3 | AR | Rho GDP-dissociation inhibitor alpha | CNS, early childhood | 1 |
| 13 | *AVIL* | 12q14.1 | AR | Advillin | SRNS | 3 |
| 14 | *CD2AP* | 6p12.3 | AD | CD2-associated protein | FSGS/SRNS, childhood, adult | 1 |
| 15 | *CD151* | 11p15.5 | AR | CD151 antigen | FSGS, Pretibial epidermolysis bullosa, deafness | 2 |
| 16 | *CFH* | 1q31.3 | AR | Complement factor H | MPGN/NS, aHUS/C3G | 1 |
| 17 | *CLCN5* | Xp11.23 | XL | Chloride voltage-gated channel 5 | Dent’s disease | 6 |
| 18 | *COL4A1* | 13q34 | AD | Collagen type 4 alpha 1 | HANAC syndrome | 8 |
| 19 | *COL4A3* | 2q36.3 | AD | Collagen type 4 alpha 3 | Alport’s syndrome/FSGS | 1 |
| 20 | *COL4A4* | 2q36.3 | AD | Collagen type 4 alpha 4 | Alport’s syndrome/FSGS | 1 |
| 21 | *COL4A5* | Xq22.3 | XL | Collagen type 4 alpha 5 | Alport’s syndrome/FSGS | 1 |
| 22 | *COQ2* | 4q21.22 | AR | Cerevisiae homolog of Q2 (Coenzyme Q10) | Mitochondrial disease + NS, encephalopathy | 1 |
| 23 | *COQ6* | 14q24.3 | AR | Cerevisiae homolog of Q6 (Coenzyme Q6) | CoQ10 deficiency, deafness | 1 |
| 24 | *COQ8B* | 19q13.2 | AR | Coenzyme Q8B | NS | 10 |
| 25 | *CRB2* | 9q33.4 | AR | Crumbs Drosophila homolog of 2, cell polarity complex component | FSGS, cerebral ventriculomegaly | 4 |
| 26 | *CTNS* | 17p13.2 | AR | Cystinosin, lysosomal cysteine transporter | FSGS | 9 |
| 27 | *CUBN* | 10p13 | AR | Cubilin | FSGS, megaloblastic anemia | 4 |
| 28 | *DAAM2* | 6p21.2 | AR | dishevelled associated activator of morphogenesis 2 | NS | 7 |
| 29 | *DGKE* | 17q22 | AR | Diacylglycerol kinase epsilon 64-KD | FSGS/aHUS | 4 |
| 30 | *E2F3* | 6p22.3 | AD | E2F transcription factor 3 | FSGS + mental retardation | 2 |
| 31 | *EMP2* | 16p13.13 | AR | Epithelial membrane protein 2 | SSNS/SRNS | 6 |
| 32 | *EYA1* | 8q13.3 | AD | EYA transcriptional coactivator and phosphatase 1 | Branchio-oto-renal syndrome | 11 |
| 33 | *FAT1* | 4q35.2 | AR | Fat tumor suppressor Drosophila, homolog of 1 | FSGS | 5 |
| 34 | *FN1* | 2q35 | AD | Fibronectin 1 | FSGS | 9 |
| 35 | *FRAS1* | 4q21.21 | AR | Fraser extracellular matrix complex subunit 1 | Fraser syndrome | 8 |
| 36 | *GLA* | Xq22.1 | XL | Galactosidase alpha | Fabry’s disease | 5 |
| 37 | *GPC5* | 13q31.3 | AR | Glypican 5 | FSGS | 12 |
| 38 | *INF2* | 14q32.33 | AD | Inverted formin 2 | FSGS | 1 |
| 39 | *ITGA3* | 17q21.33 | AR | Integrin alpha-3 | Congenital lung disease + NS + epidermolysis bullosa | 4 |
| 40 | *ITGB4* | 17q25.1 | AR | Integrin beta-4 | Epidermolysis bullosa + FSGS, pyloric atresia | 1 |
| 41 | *KANK1* | 9p24.3 | AR | KN motif and Ankyrin repeat domain containing protein 1 | SSNS, adult, intellectual disability | 4 |
| 42 | *KANK2* | 19p13.2 | AR | KN motif and Ankyrin repeat domain containing protein 2 | SSNS/SDNS + hematuria | 2 |
| 43 | *KANK4* | 1p31.3 | AR | KN motif and Ankyrin repeat domain containing protein 4 | SRNS + hematuria, intellectual disability, facial dysmorphism, ASD | 4 |
| 44 | *LAGE3* | Xq28 | XL | L antigen family member 3 | Galloway-Mowat syndrome 2 (FSGS) | 3 |
| 45 | *LAMA5* | 20q13.33 | AR? | Laminin subunit alpha 5 | FSGS, adult | 1 |
| 46 | *LAMB2* | 3p21.31 | AR | Laminin beta-2 | Pierson syndrome | 1 |
| 47 | *LAMB3* | 1q32.2 | AR | Laminin beta-3 | Epidermolysis bullosa, junctional | 13 |
| 48 | *LMNA* | 1q22 | AD | Lamin A, Lamin C | FSGS, Partial lipodystrophy | 1 |
| 49 | *LMX1B* | 9q33.3 | AD | LIM hoemobox transcription factor 1 beta | FSGS, Nail-Patella syndrome | 1 |
| 50 | *MAFB* | 20q12 | AD | MAF bZIP transcription factor B | Multicentric Carpotarsal Osteolysis Syndrome (MCOS), Duane retraction syndrome 3 | 14 |
| 51 | *MAGI2* | 7q21.11 | AR | Membrane-associated Guanylate kinase, WW and PDZ domains-containing 2 | NS | 3 |
| 52 | *MTTL1* | M | M | Transfer RNA. Mitochondrial, leucine 1 | MELAS syndrome, adult | 4 |
| 53 | *MTTL2* | M | M | Transfer RNA. Mitochondrial, leucine 2 | MELAS syndrome | 11 |
| 54 | *MUC1* | 1q22 | AD | Mucin 1, cell surface associated | ADTKD 2 | 16 |
| 55 | *MYH9* | 22q12.3 | AD | Myosin heavy chain 9 | MYH-related disorders (Epstein syndrome, Fechtner syndrome) | 1 |
| 56 | *MYO1E* | 15q22.2 | AR | non-muscle Myosin 1E | SRNS | 1 |
| 57 | *NEIL1* | 15q24.2 | AR | Nei like DNA glycosylase 1 |  | 1 |
| 58 | *NPHS1* | 19q13.12 | AR | Nephrin | CNS/FSGS/SRNS | 1 |
| 59 | *NPHS2* | 1q25.2 | AR | Podocin | CNS/FSGS/SRNS | 1 |
| 60 | *NUP93* | 16q13 | AR | Nucleoporin 93-KD | Childhood SRNS | 4 |
| 61 | *NUP107* | 12q15 | AR | Nucleoporin 107-KD | Childhood SRNS, microcephaly | 4 |
| 62 | *NUP160* | 11p11.2 | AR | Nucleoporin 160-KD | Childhood SRNS | 15 |
| 63 | *NUP205* | 7q33 | AR | Nucleoporin 205-KD | Childhood SRNS | 4 |
| 64 | *NXF5* | Xq22.1 | XL | Nuclear RNA export factor 5 | FSGS + heart block disorder | 1 |
| 65 | *OCRL* | Xq26.1 | XL | Phosphatidylinositol bisphophate-5-phosphatase | Dent disease + FSGS | 6 |
| 66 | *OSGEP* | 14q11.2 | AR | O-sialoglycoprotein endopeptidase | Galloway-Mowat syndrome 3 (FSGS) | 3 |
| 67 | *PAX2* | 10q24.31 | AD | Paired box 2 | Renal-Coloboma syndrome | 5 |
| 68 | *PDSS2* | 6q21 | AR | Prenyl diphosphate synthase, subunit 2 | Leigh syndrome, encephalomyopathy | 1 |
| 69 | *PLCE1* | 10p23.33 | AR | Phospholipase C epsilon-1 | CNS/SRNS, early-onset | 1 |
| 70 | *PMM2* | 16p13.2 | AR | Phosphomannomutase 2 | FSGS, childhood | 1 |
| 71 | *PODXL* | 7q32.3 | AD | Podocalyxin like protein 1 | FSGS | 1 |
| 72 | *PTPRO (GLEPP1)* | 12p12.3 | AR | Protein-tyrosine phosphate receptor-type O | FSGS, childhood | 1 |
| 73 | *RCAN1* | 21q22.12 | AD | regulator of calcineurin 1 | Down syndrome? | 17 |
| 74 | *SCARB2* | 4q21.1 | AR | Lysosomal integral membrane protein 2 | Action myoclonus renal failure syndrome | 1 |
| 75 | *SGPL1* | 10q22.1 | AR | Sphingosine-1-phosphate lyase 1 | FSGS, childhood, hypogonadism, adrenal insufficiency | 3 |
| 76 | *SMARCAL1* | 2q35 | AR | SWI/SNF-related matrix-associated actin-dependent regulator of chromatin, subfamily A like protein 1 | Schimke immune-osseous dysplasia | 1 |
| 77 | *SYNPO* | 5q33.1 | AD | Synaptopodin | Sporadic FSGS | 1 |
| 78 | *TP53RK* | 20q13.12 | AR | TP53-regulating kinase | Galloway-Mowat syndrome 4 (FSGS) | 3 |
| 79 | *TPRKB* | 2p13.1 | AR | TP53RK- binding protein | Galloway-Mowat syndrome 5 (FSGS) | 3 |
| 80 | *TRPC6* | 11q22.1 | AD | Trasient receptor potential cation channel subfamily C member 6 | FSGS, adulthood, rarely childhood | 1 |
| 81 | *TTC21B* | 2q24.3 | AR | Tetratricopeptide repeat domain-containing protein 21B | FSGS + tubulointerstitial involvement | 5 |
| 82 | *UMOD* | 16p12.3 | AD | Uromodulin | ADTKD, Medullary cystic kidney disease | 6 |
| 83 | *WDR19* | 4p14 | AR | WD (tryptophan-aspartic acid) repeat domain 19 | Senior-Loken syndrome | 9 |
| 84 | *WDR73* | 15p25.2 | AR | WD (tryptophan-aspartic acid) repeat domain 73 | Galloway-Mowat syndrome (FSGS) | 4 |
| 85 | *WT1* | 11p13 | AD | Wilms tumor 1 | FSGS, Denys-Drash syndrome, Frasier syndrome | 1 |
| 86 | *XPO5* | 6p21.1 | AR | Exportin 5 | Childhood SRNS | 4 |
| 87 | *ZEB1* | 10p11.2 | AR | Zinc finger E-box binding homeobox 1 | Posterior Polymorphous Corneal Dystrophy 3, Fuchs Endothelial Corneal Dystrophy 6 | 1 |
| 88 | *ZMPSTE24* | 1p34.2 | AR | Zinc metalloprotease STE 24 | Mandibuloacral dysplasia + FSGS | 1 |

AD = autosomal dominant, ADTKD = autosomal dominant tubulointerstitial kidney diseases, aHUS = atypical hemolytic uremic syndrome, AR = autosomal recessive, CNS = congenital nephrotic syndrome, FSGS = focal segmental glomerulosclerosis, HANAC syndrome = Hereditary angiopathy with nephropathy, aneurysms, and muscle cramps syndrome, M = mitochondrial, MELAS syndrome = Mitochondrial Encephalopathy, Lactic Acidosis, and Stroke-like episodes syndrome, MPGN = membranoproliferative glomerulonephritis, SRNS = steroid-resistant nephrotic syndrome, SSNS = steroid-sensitive nephrotic syndrome, XL = X-linked

**References for Supplementary Table S1**

1. Gast, C., Pengelly, R. J., Lyon, M., *et al*. Collagen (COL4A) mutations are the most frequent mutations underlying adult focal segmental glomerulosclerosis. *Nephrol Dial Transplant*. **31**, 961-970 (2016).
2. Sen, E.S., Dean, P., Yarram-Smith, L., *et al*. Clinical genetic testing using a custom-designed steroid-resistant nephrotic syndrome gene panel: analysis and recommendations. *J Med Genet*. **54**, 795-804 (2017).
3. Sharif, B. & Barua, M. Advances in molecular diagnosis and therapeutics in nephrotic syndrome and focal and segmental glomerulosclerosis. *Curr Opin Nephrol Hypertens*. **27**, 194-200 (2018).
4. Lepori, N., Zand, L., Sethi, S., *et al*. Clinical and pathological phenotype of genetic causes of focal segmental glomerulosclerosis in adults. *Clin Kidney J*. **11**, 179-190 (2018).
5. Harita, Y. Application of next-generation sequencing technology to diagnosis and treatment of focal segmental glomerulosclerosis. *Clin Exp Nephrol*. **22**, 491-500 (2018).
6. Yao, T., Udwan, K., John, R., *et al*. Integration of genetic testing and pathology for the diagnosis of adults with FSGS. *Clin J Am Soc Nephrol*. **14**, 213-223 (2019).
7. Schneider, R., Deutsch, K., Hoeprich, GJ., *et al*. DAAM2 Variants Cause Nephrotic Syndrome via Actin Dysregulation. *Am J Hum Genet*. **107**, 1113-1128 (2020).
8. Savige, J. & Harraka, P. Pathogenic Variants in the Genes Affected in Alport Syndrome (COL4A3-COL4A5) and Their Association With Other Kidney Conditions: A Review. *Am J Kidney Dis*. **78**, 857-864 (2021).
9. Warejko, J.K., Tan, W., Daga, A., *et al*. Whole exome sequencing of patients with steroid-resistant nephrotic syndrome. *Clin J Am Soc Nephrol*. **13**, 53-62 (2018).
10. Maeoka, Y., Doi, T., Aizawa, M., *et al*. A case report of adult-onset COQ8B nephropathy presenting focal segmental glomerulosclerosis with granular swollen podocytes. *BMC Nephrol*. **21**, 376 (2020).
11. De Vriese, A. S., Sethi, S., Nath, K. A., Glassock, R.J. & Fervenza, F.C. Differentiating primary, genetic, and secondary FSGS in adults: A Clinicopathologic Approach. *J Am Soc Nephrol*. **29**, 759-774 (2018).
12. Okamoto, K., Tokunaga, K., Doi, K., *et al*. Common variation in GPC5 is associated with acquired nephrotic syndrome. *Nat Genet*. **43**, 459-463 (2011).
13. Hata, D., Miyazaki, M., Seto, S., *et al*. Nephrotic syndrome and aberrant expression of laminin isoforms in glomerular basement membranes for an infant with Herlitz junctional epidermolysis bullosa. *Pediatrics*. **116**, e601-e607 (2005).
14. Kaimori JY, Mori T, Namba-Hamano T, *et al*. Cyclosporine A Treatment of Proteinuria in a New Case of MAFB-Associated Glomerulopathy without Extrarenal Involvement: A Case Report. *Nephron*. **145**, 445-450 (2021).
15. Zhao, F., Zhu, JY., Richman, A., *et al*. Mutations in *NUP160* Are Implicated in Steroid-Resistant Nephrotic Syndrome. *J Am Soc Nephrol*. **30**, 840-853 (2019).
16. Trimarchi, H., Paulero, M., Rengel, T., *et al*. Mucin-1 Gene Mutation and the Kidney: The Link between Autosomal Dominant Tubulointerstitial Kidney Disease and Focal and Segmental Glomerulosclerosis. *Case Rep Nephrol*. 9514917 (2018).
17. Lane, BM., Murray, S., Benson, K., *et al*. A Rare Autosomal Dominant Variant in Regulator of Calcineurin Type 1 (*RCAN1*) Gene Confers Enhanced Calcineurin Activity and May Cause FSGS. *J Am Soc Nephrol*. 32, 1682–1695 (2021).

**Supplementary Table S2: Detailed Clinical, laboratory, and pathological characteristics of FSGS patients in our cohort**

| **Categories** | **Subcategories** | **Number of Patients (%)** |
| --- | --- | --- |
| *Clinical characteristics* | | |
| Sex | Male | 28/53 (52.8%) |
| Family history of kidney diseases | Positive | 2/53 (3.8%) |
| Extra-renal syndromic manifestation | Positive | 1/53 (1.9%) |
| Age at kidney biopsy | Age 1-5 years | 6/53 (11.3%) |
|  | Age 6-18 years | 12/53 (22.6%) |
|  | Age 19-45 years | 19/53 (35.9%) |
|  | Age 45-60 years | 13/53 (24.5%) |
|  | Age > 60 years | 3/53 (5.7%) |
| Clinical diagnosis/syndrome | SSNS | 5/53 (9.4%) |
|  | SRNS | 31/53 (58.5%) |
|  | Proteinuric CKD | 17/53 (32.1%) |
| Immunosuppressive medications received | Prednisolone | 40/53 (75.5%) |
|  | Cyclosporin | 17/53 (32.1%) |
|  | Tacrolimus | 6/53 (11.3%) |
|  | Cyclophosphamide | 8/53 (15.1%) |
|  | Mycophenolate mofetil | 4/53 (7.5%) |
|  | Others | 2/53 (5.6%) |
|  | No immunosuppressive received | 13/53 (24.5%) |
| Mean time of follow-up | | 9.5 years |
| Current status | No proteinuria and normal creatinine | 4/53 (7.5%) |
|  | Proteinuric CKD | 16/53 (67.9%) |
|  | ESRD | 12/53 (22.7%) |
|  | Dead | 1/53 (1.9%) |
| RRT mode | Hemodialysis | 5/12 (41.7%) |
|  | Peritoneal dialysis | 5/12 (41.7%) |
|  | Kidney transplantation | 2/12 (16.6%) |
| Median time from FSGS diagnosis to ESRD | | 8 years |
| *Pathological characteristics (Light microscopy)* | | |
| Columbia classification of FSGS | Tip lesion | 11/53 (20.7%) |
|  | Hilar/Perihilar lesion | 4/53 (7.5%) |
|  | Cellular variant | 2/53 (3.8%) |
|  | Collapsing variant | 2/53 (3.8%) |
|  | Not otherwise specified (NOS) | 34/53 (64.2%) |
| IFTA | No IFTA | 22/53 (41.5%) |
|  | Mild IFTA | 22/53 (41.5%) |
|  | Moderate IFTA | 5/53 (9.5%) |
|  | Marked/Diffused IFTA | 4/53 (7.5%) |
| Vessels | Unremarkable | 35/53 (66.0%) |
|  | Hyaline change or fibrous thickening | 18/53 (34.0%) |
| *Pathological characteristics (Immunofluorescent study)* | | |
| Negative or non-specific IF staining | | 17/42 (40.5%) |
| Only segmental IgM and/or C3 staining | | 11/42 (26.2%) |
| Mesangial IgM and/or C3 staining | | 12/42 (28.5%) |
| Others | | 2/42 (4.8%) |
| *Pathological characteristics (Electron microscopy)* | | |
| Podocyte foot process effacement | No | 0/18 (0%) |
|  | Focal | 12/18 (66.7%) |
|  | Diffused | 6/18 (33.3%) |
| Microvillous transformation | No | 2/18 (11.1%) |
|  | Focal | 11/18 (61.1 %) |
|  | Diffused | 5/18 (27.8%) |
| Irregular GBM | Yes | 10/18 (55.6%) |
| Multilayering of GBM | Yes | 0/18 (0%) |
| Electron dense deposits | Positive | 1/18 (5.6%) |

CKD = chronic kidney disease, ESRD = end-stage renal disease, IF = immunofluorescent study, IFTA = interstitial fibrosis and tubular atrophy, IgM = immunoglobulin M, EM = electron microscopy, GBM = glomerular basement membrane, FSGS = focal segmental glomerulosclerosis, LM = light microscopy, RRT = renal replacement therapy, SRNS = steroid-resistant nephrotic syndrome, SSNS = steroid-sensitive nephrotic syndrome

**Supplementary Table S3: Variant interpretation of pathogenic or likely pathogenic variants**

|  | **Patient 1**  **G2000207** | **Patient 2**  **G7372** | **Patient 3**  **G7383** | **Patient 4**  **G7391** | **Patient 5**  **G7411** | **Patient 6**  **G7525** |
| --- | --- | --- | --- | --- | --- | --- |
| Gene | *CLCN5* | *LMX1B* | *COL4A4* | *COL4A4* | *COL4A4* | *MAFB* |
| Inheritance | AD | AD | AD | AD | AD | AD |
| Chromosome  (HG19) | [chrX:49855147](http://grch37.ensembl.org/Homo_sapiens/Location/View?db=core;r=X:49855147-49855147) | [chr9:129455598](http://grch37.ensembl.org/Homo_sapiens/Location/View?db=core;r=9:129455598-129455598) | [chr2:227942792](http://grch37.ensembl.org/Homo_sapiens/Location/View?db=core;r=2:227942792-227942792) | [chr2:227919418](http://grch37.ensembl.org/Homo_sapiens/Location/View?db=core;r=2:227919418-227919418) | [chr2:227967530](http://grch37.ensembl.org/Homo_sapiens/Location/View?db=core;r=2:227967530-227967530) | [chr20:39317353](http://grch37.ensembl.org/Homo_sapiens/Location/View?db=core;r=20:39317353-39317353) |
| NM | [NM_001127898.3](https://www.ncbi.nlm.nih.gov/nuccore/NM_001127898.3) | [NM_001174146.1](https://www.ncbi.nlm.nih.gov/nuccore/NM_001174146.1) | [NM_000092.4](https://www.ncbi.nlm.nih.gov/nuccore/NM_000092.4) | [NM_000092.4](https://www.ncbi.nlm.nih.gov/nuccore/NM_000092.4) | [NM_000092.4](https://www.ncbi.nlm.nih.gov/nuccore/NM_000092.4) | [NM_005461.4](https://www.ncbi.nlm.nih.gov/nuccore/NM_005461.4) |
| Type of mutation | Nonsense | Missense | Missense | Missense | Frameshift indels | Nonsense |
| Known/Novel | Known [14] | Known [15] | Known [16,17] | Known [18,19] | Novel | Novel |
| Variant | c.2119C>T  (p.Arg707Ter) | c.737G>A  (p.Arg246Gln) | c.1805G>A  (p.Gly602Glu) | c.2752G>A  (p.Gly918Arg) | c.905delG  (p.Gly302ValfsTer23) | c.138C>A  (p.Cys46Ter) |
| Original AA group | Positively charged | Positively charged | Non-polar aliphatic | Non-polar aliphatic | Non-polar aliphatic | Polar, uncharged |
| Changed AA group | Stop codon | Polar uncharged | Negatively charged | Positively charged | N/A | Stop codon |
| AF (Thailand database) | 0 | 0 | 0 | 0 | 0 | 0 |
| AF (gnomAD, whole population) | 0 | 0 | 0 | 9/280892 =  0.00003185 | 0 | 0 |
| AF (gnomAD, EA population) | 0 | 0 | 0 | 2/19530 =  0.0001024 | 0 | 0 |
| M-CAP prediction | N/A | 0.813  (Pathogenic) | 0.893  (Pathogenic) | 0.897  (Pathogenic) | N/A | N/A |
| Polyphen-2 prediction | N/A | 1  (Damaging) | 1  (Damaging) | 1  (Damaging) | N/A | N/A |
| PROVEAN prediction | N/A | -3.91  (Deleterious) | -7.64  (Deleterious) | -7.17  (Deleterious) | N/A | N/A |
| Sift prediction | N/A | 0  (Damaging) | 0  (Damaging) | 0  (Damaging) | N/A | N/A |
| Variance conclusion | Pathogenic | Likely Pathogenic | Likely pathogenic | Likely pathogenic | Likely pathogenic | Likely pathogenic |
| ACMG criteria | PVS1+PM2+PM4 | PS3+PM2+PP3 | PM1+PM2  +PP3+PP5 | PM1+PM2  +PP3+PP5 | PVS1+PM2 | PVS1+PM2 |

AA = amino acid, ACMG = American College of Medical Genetics and Genomics, AD = autosomal dominant, AF = allele frequency, EA = east Asia, FSGS = focal segmental glomerulosclerosis, M-CAP = Mendelian clinically applicable pathogenicity score, N/A = not applicable, Polyphen-2 = polymorphism phenotyping v.2, PROVEAN = protein variation effect analyzer, Sift = sorting intolerance from tolerance

**Supplementary Table S4: Patients with variants with uncertain significance (VUS)**

| **Patients** | **Sex** | **Age at FSGS diagnosis (years)** | **Clinical presentation** | **Current status** | **Gene** | **Inheritance** | **Variance** | **Protein** | **Allele frequency**  **(Thai database)** | **Allele frequency**  **(gnomAD, East Asian)** | **Computational model*** |
| --- | --- | --- | --- | --- | --- | --- | --- | --- | --- | --- | --- |
| G1800276 | F | 5 | SRNS | Proteinuric CKD | *ARHGAP24* | AD | c.1190G>A | p.(Gly397Asp) | 0.0078 | 0.0012 | Conflicting results |
| G2100488 | F | 91 | SRNS | Proteinuric CKD | *ARHGAP24* | AD | c.1875T>G | p.(Ser625Arg) | 0 | 0 | Pathogenic/Damaging |
| G6458 | M | 5 | SRNS | Asymptomatic proteinuria | *ANLN* | AD | c.1760T>A | p.(Met587Lys) | 0.0018 | 0 | Conflicting results |
| G6787 | F | 8 | SRNS | Asymptomatic proteinuria | *DAAM2* | AR | c.602T>C | p.(Ile201Thr) | 0.0009 | 0.00006 | Pathogenic/Damaging |
|  |  |  |  |  | *DAAM2* | AR | c.803G>A | p.(Arg268Gln) | 0 | 0.0003 | Pathogenic/Damaging |
|  |  |  |  |  | *INF2* | AD | c.2255G>A | p.(Arg752His) | 0.0009 | 0.0002 | Benign/Tolerated |
| G6914 | M | 56 | SRNS | ESRD on HD | *FN1* | AD | c.3187A>G | p.(Thr1063Ala) | 0 | 0.002 | Conflicting results |
| G7294 | M | 13 | SRNS | Asymptomatic proteinuria | *ANLN* | AD | c.1462G>A | p.(Val488Ile) | 0 | 0.0003 | Benign/Tolerated |
| G7384 | F | 35 | SSNS | No proteinuria and normal creatinine | *LMNA* | AD | c.1279C>T | p.(Arg427Cys) | 0 | 0.0001 | Conflicting results |
|  |  |  |  |  | *SYNPO* | AD | c.2206G>C | p.(Glu736Gln) | 0 | 0 | Conflicting results |
| G7392 | F | 42 | Proteinuric CKD | ESRD on PD | *FN1* | AD | c.5577C>G | p.(Ile1859Met) | 0 | 0 | Benign/Tolerated |
|  |  |  |  |  | *ACSL4* | AD | c.1007A>G | p.(Tyr336Cys) | 0 | 0.0002 | Conflicting results |
| G7393 | F | 54 | SSNS | Asymptomatic proteinuria | *MYH9* | AD | c.4391G>A | p.(Arg1464His) | 0 | 0.0006 | Pathogenic/Damaging |
| G7420 | F | 41 | SRNS | Proteinuric CKD | *LMX1B* | AD | c.1073C>T | p.(Ser358Phe) | 0 | 0.0003 | Conflicting results |
| G7535 | F | 50 | Asymptomatic proteinuria with normal renal function | Asymptomatic proteinuria | *ANLN* | AD | c.659T>A | p.(Phe220Tyr) | 0 | 0.0001 | Benign/Tolerated |
|  |  |  |  |  | *MYH9* | AD | c.1108+7C>T | N/A | 0 | 0.0003 | N/A |
| G7571 | M | 50 | SRNS | Proteinuric CKD | *TRPC6* | AD | c.2084A>G | p.(Tyr695Cys) | 0 | 0.0001 | Conflicting results |
| G7614 | M | 33 | SSNS | No proteinuria and normal creatinine | *PAX2* | AD | c.541C>T | p.(Pro181Ser) | 0 | 0 | Conflicting results |
| G7619 | F | 10 | SRNS | Asymptomatic proteinuria | *ALMS1* | AR | c.249C>A | p.(His84Gln) | 0 | 0.001 | Conflicting results |
|  |  |  |  |  | *ALMS1* | AR | c.4246C>T | p.(Arg1418Trp) | 0 | 0.002 | Conflicting results |
| G7702 | M | 3 | SRNS | ESRD on HD | *FN1* | AD | c.989A>G | p.(Gln330Arg) | 0 | 0 | Conflicting results |
|  |  |  |  |  | *MYH9* | AD | c.519-4G>A | N/A | 0 | 0.00005 | N/A |

CKD = chronic kidney disease, ESRD = end-stage renal disease, HD = hemodialysis, N/A = not applicable, PD = peritoneal dialysis, SRNS = steroid-resistant nephrotic syndrome, SSNS = steroid-sensitive nephrotic syndrome

* We used four computational models including M-CAP, Polyphen-2, PROVEAN and Sift. Conflicting results mean there are conflicting results between these four models. Pathogenic/Damaging and Benign/Tolerated means all of four computational models provided the same results.

**Supplementary Figure S1: Pedigrees of two patients with a family history of renal diseases**


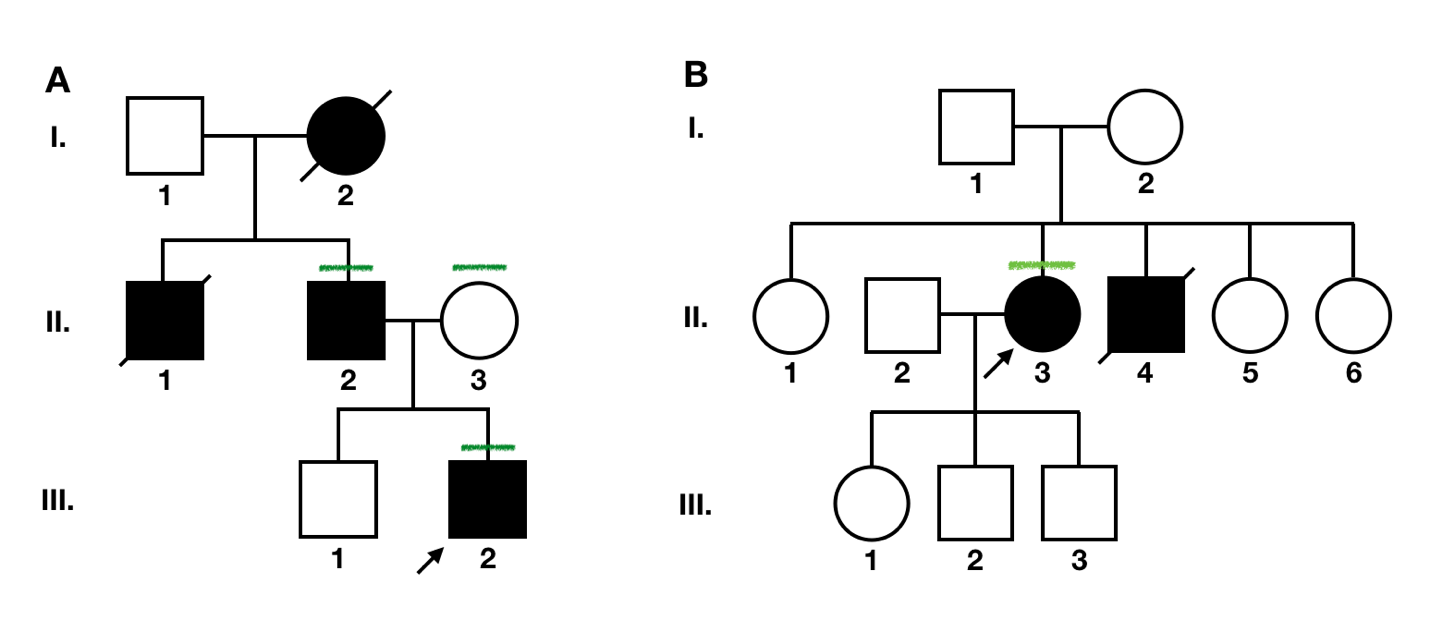


S1A: Patient 2 had strong family history of renal diseases. Patient 2 himself presented with SRNS, but his father presented with advanced stage CKD. This family had known missense mutation in *LMX1B* gene (c.737G>A, p.Arg246Gln), which was known to have intense phenotypic variability within and between a family.

S1B: Patient 5 had a younger brother with history of renal disease and dialysis. However, he died before patient 5 was offered genetic testing. Patient 5 had deletion in *COL4A4* leading to frameshift mutation and premature stop codon (*COL4A4*, c.905delG, p.Gly302ValfsTer32). Genetic mutation in this family cannot be proven to be familial or *de novo*.

Mutation analysis was performed in patients with green bar.

CKD = chronic kidney disease, FSGS = focal segmental glomerulosclerosis, SRNS = steroid-resistant nephrotic syndrome
